# Dual-Channel Extrusion-Based 3D Printing of a Gradient Hydroxyapatite Hydrogel Scaffold with Spatial Curved Architecture

**DOI:** 10.3390/gels12010093

**Published:** 2026-01-21

**Authors:** Yahao Wang, Yongteng Song, Qingxi Hu, Haiguang Zhang

**Affiliations:** 1 Rapid Manufacturing Engineering Center, School of Mechatronical Engineering and Automation, Shanghai University, Shanghai 200444, China; 2 National Demonstration Center for Experimental Engineering Training Education, Shanghai University, Shanghai 200444, China; 3 Shanghai Key Laboratory of Intelligent Manufacturing and Robotics, Shanghai University, Shanghai 200072, China; 4 School of Mechanical and Aerospace Engineering, Nanyang Technological University, Singapore 639798, Singapore

**Keywords:** cartilage scaffold, continuous hydroxyapatite gradient, dual-channel extrusion 3D printing, biomimetic curved architecture, tissue engineering hydrogels

## Abstract

A biomimetic cartilage scaffold featuring a continuous hydroxyapatite (HA) concentration gradient and a spatially curved architecture was developed using a dual-channel mixing extrusion-based 3D printing approach. By dynamically regulating the feeding rates of two bioinks during printing, a continuous HA gradient decreasing from the bottom to the top of the scaffold was precisely achieved, mimicking the compositional transition from the calcified to the non-calcified cartilage region in native articular cartilage. The integration of gradient material deposition with synchronized multi-axis motion enabled accurate fabrication of curved geometries with high structural fidelity. The printed scaffolds exhibited stable swelling and degradation behavior and showed improved compressive performance compared with step-gradient counterparts. Rheological analysis confirmed that the bioinks possessed suitable shear-thinning and recovery properties, ensuring printability and shape stability during extrusion. In vitro evaluations demonstrated good cytocompatibility, supporting bone marrow mesenchymal stem cell (BMSC) adhesion and proliferation. Chondrogenic assessment based on scaffold extracts indicated that the incorporation of HA and its gradient distribution did not inhibit cartilage-related extracellular matrix synthesis, confirming the biosafety of the composite hydrogel system. Overall, this study presents a controllable and versatile fabrication strategy for constructing curved, compositionally graded cartilage scaffolds, providing a promising platform for the development of biomimetic cartilage tissue engineering constructs.

## 1. Introduction

Articular cartilage is a highly specialized connective tissue that plays a vital role in maintaining smooth joint articulation, distributing loads, and providing a nearly frictionless gliding surface. Despite its critical importance, cartilage is avascular, aneural, and has a very limited capacity for self-repair once injured [1,2]. Even small defects often progress into larger lesions, eventually leading to osteoarthritis. The clinical burden of cartilage damage is therefore substantial, affecting millions worldwide. Current surgical interventions—such as microfracture, autologous chondrocyte implantation, and osteochondral grafting—may alleviate symptoms temporarily, but rarely restore full structural and functional integrity of native cartilage [3,4,5].

Tissue engineering scaffolds have emerged as promising alternatives, aiming to provide a biomimetic extracellular matrix (ECM) environment for cartilage regeneration. A wide variety of natural polymers, synthetic polymers, ceramics, and composites have been employed to fabricate scaffolds that support chondrocyte proliferation and ECM deposition [6]. Although recent studies have reported scaffolds with compositional or mineral gradients, many designs remain relatively simple—often flat, cylindrical, or block-shaped—and thus fail to simultaneously reproduce two critical features of native articular cartilage: its curved surface geometry and the continuous mineral gradient across the cartilage layer [7,8,9].

The geometry of articular cartilage is curved, conforming to joint surface contours. This curvature is essential for joint biomechanics: it helps distribute compressive and shear forces more evenly, reducing stress concentrations that accelerate degeneration, while also improving lubrication and articulation stability [10,11,12]. Recent advances in multi-axial 3D bioprinting have enabled the fabrication of curved scaffolds with higher fidelity to native joint morphology [13]. However, such designs typically employed uniform material composition in the cartilage phase and did not address the mineral gradient essential for the calcified cartilage transition.

Equally important, native cartilage exhibits a depth-dependent compositional gradient. From the superficial zone to the calcified cartilage, extracellular matrix composition changes significantly, with hydroxyapatite (HA) content gradually increasing near the cartilage–bone interface [12,14]. This mineral gradient provides mechanical compatibility between soft cartilage and rigid subchondral bone, reduces stress discontinuities, and minimizes delamination risks, while enhancing interfacial stability under repetitive loading.

In recent years, gradient scaffold designs have been investigated to improve tissue regeneration by more closely mimicking the continuous compositional variation in native tissues. For example, Liu et al. fabricated a chirality-controlled HA gradient scaffold and demonstrated its effectiveness in promoting osteochondral regeneration via mechanotransduction pathways, showing enhanced biological performance in vivo and in vitro compared with non-gradient controls [15]. Similarly, Xu et al. reported a nano-hybrid gradient scaffold with in situ generated nano-hydroxyapatite (nHAP) distributed continuously throughout the construct, and this gradient scaffold was shown to promote bone marrow mesenchymal stem cell proliferation and better induce articular cartilage regeneration in defect models, suggesting the potential of HA gradient distributions in articular repair applications [16]. These studies illustrate that spatially varied HA distributions within scaffolds can modulate cellular responses and tissue formation, highlighting the importance of controlled HA gradients in scaffold design for complex tissue engineering. In addition to these studies, several independent works have explored cartilage-related gradient scaffolds using different material systems or fabrication strategies, further highlighting the importance of controlled compositional gradients in regulating chondrogenic responses and tissue formation [17,18].

Despite these advances, increasing attention has been paid to reproducing region-specific features in osteochondral scaffold design, and multiple fabrication strategies have been developed to create distinct functional zones within a single construct. A common approach is the construction of multilayered scaffolds, in which different regions are defined by discrete layers with tailored material compositions or properties, thereby representing cartilage, calcified cartilage, and subchondral bone, respectively [19,20,21]. Alternatively, some designs generate transitional regions through partial overlap between adjacent layers, achieved by sequential extrusion [22], alternating deposition [23], or the combination of extrusion-based printing with casting techniques, where the overlapping zone is designated as the calcified cartilage layer [24,25,26]. In addition, mixed interfacial regions have been created using field-assisted approaches—such as gravity-driven settling or magnetic field guidance—to induce material redistribution between layers, resulting in an intermediate zone with gradually mixed characteristics [27].

While these strategies enable the formation of regionally distinct architectures, they are predominantly implemented as discrete layers or partially mixed interfaces rather than as precisely controlled, continuous gradients. Moreover, most existing designs have been developed in the context of bone or osteochondral repair, where gradient concepts are primarily employed to enhance interfacial integration or osteogenic performance. In contrast, the application of controlled compositional gradients within cartilage-specific scaffolds—particularly to mimic the gradual transition from calcified cartilage to non-calcified cartilage—remains relatively underexplored.

At the same time, scaffold geometry has largely been investigated independently of gradient design. Curved or anatomically conformal scaffolds have been fabricated to better match joint surface morphology, yet these constructs typically employ homogeneous material compositions within the cartilage phase. Conversely, HA-gradient scaffolds were often designed with simplified geometries, such as flat or block-shaped structures, assuming that geometric effects were secondary to compositional control [28,29]. As a result, the combined influence of continuous mineral gradients and curved surface geometry within a cartilage-specific scaffold remains insufficiently explored.

In the present study, we propose a novel cartilage scaffold that integrates a curved morphology with an HA concentration gradient. The scaffold was fabricated via multi-axial extrusion-based 3D printing, enabling precise control over both geometry and material distribution. In this design, the HA content progressively decreases from the bottom to the top, mimicking the compositional transition from calcified cartilage to the superficial cartilage zone, while the curved surface conforms to the native joint morphology. Based on this concept, a continuously graded HA hydrogel scaffold (CG-HA) was designed as the primary focus of this study to achieve a smooth HA concentration transition, whereas a stepwise HA scaffold (S-HA), composed of discrete layers with different HA contents, was fabricated solely as a control for comparison. Correspondingly, a series of gelatin methacryloyl (GelMA)–sodium alginate (SA)-based bioinks (GS) with different HA contents were prepared using phosphate-buffered saline (PBS) as the solvent, including GS-HA0, GS-HA1.5, and GS-HA3, to facilitate gradient formation during the printing process. This dual-biomimetic strategy is expected to address the limitations of previous scaffold designs and provide a promising approach for cartilage tissue engineering and repair.

## 2. Results and Discussion

### 2.1. Morphology and Structural Characterization

**Figure 1:**
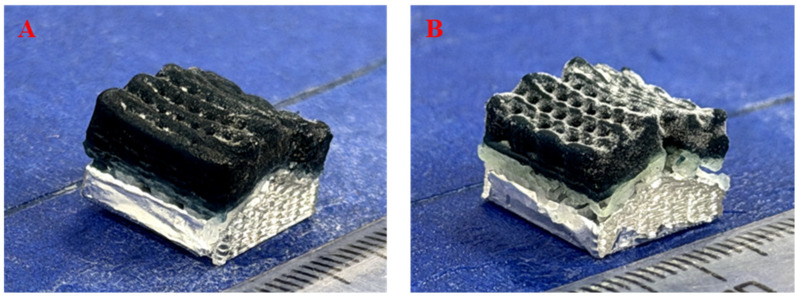
Macroscopic morphology and HA gradient visualization of CG-HA and S-HA scaffolds. (**A**) CG-HA. (**B**) S-HA.

The printed scaffolds exhibited smooth and continuous filament deposition with well-defined layer stacking, demonstrating high printing accuracy and structural fidelity. As shown in Figure 1, both the CG-HA Scaffold and the S-HA Scaffold preserved their designed spatially curved architectures without noticeable collapse or deformation after printing and subsequent crosslinking, confirming both the precision of the dual-channel extrusion process and the structural stability of the hydrogel network, which is essential for maintaining the biomimetic cartilage geometry. To visually indicate the distribution of HA, a blue dye was incorporated into the GS-HA0 Bioink, allowing the HA gradient to be represented by gradual color variation. In the CG-HA Scaffold, the blue color gradually deepened from bottom to top, indicating a progressive decrease in HA content and confirming the successful fabrication of a continuous HA concentration gradient, which mimics the natural transition from the calcified cartilage zone to the non-calcified cartilage region in native articular cartilage.

In contrast, for the S-HA Scaffold, a green dye was added to the GS-HA1.5 Bioink to distinguish different compositional layers. As shown in Figure 1, the S-HA Scaffold displayed distinct color stratification, with a white bottom layer, a green intermediate layer, and a blue top layer. The sharp color boundaries between adjacent layers indicate discrete changes in HA content rather than a gradual transition, highlighting the difference between stepwise and continuous gradient designs.

**Figure 2:**
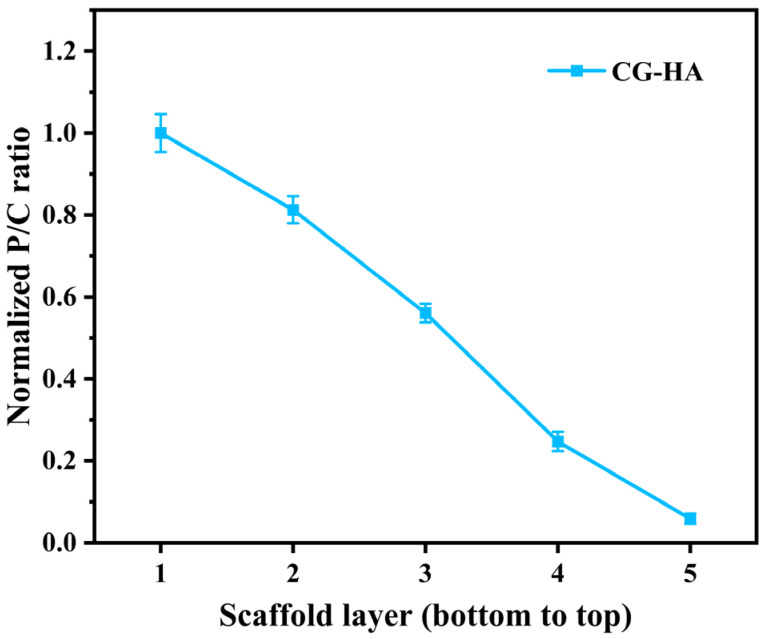
Semi-quantitative EDS analysis of the HA gradient within the CG-HA scaffold based on the normalized P/C ratio across different scaffold layers (bottom to top).

To further verify the distribution of HA along the scaffold depth, energy-dispersive X-ray spectroscopy (EDS) analysis was performed on the CG-HA scaffold at representative positions from the bottom to the top regions of the scaffold. As shown in Figure 2, the normalized phosphorus-to-carbon (P/C) ratio exhibited a clear depth-dependent variation across different scaffold layers.

Specifically, the normalized P/C ratio was highest in the bottom region of the scaffold (Layer 1, corresponding to 3% (*w*/*v*) HA) and gradually decreased toward the top region (Layer 5, corresponding to 0% (*w*/*v*) HA). This continuous downward trend in normalized P/C values is consistent with the designed HA concentration gradient, in which the mineral content decreases progressively along the scaffold height. These results provide semi-quantitative compositional evidence confirming the successful fabrication of a continuous HA gradient within the scaffold, and further demonstrate the effective establishment of a depth-dependent mineral distribution along the curved architecture. It should be noted that a baseline P signal was still detected in the top layer where the designed HA content was 0% (*w*/*v*), which can be attributed to phosphate ions originating from the PBS solvent used in the bioinks. Therefore, the normalized P/C ratio reflects a relative reduction in mineral content rather than its absolute absence. Such compositional continuity and gradient variation are important for mimicking the native cartilage microenvironment and may contribute to improved mechanical performance and biological compatibility of the scaffold. Moreover, the spatially curved architecture, inspired by native articular cartilage geometry, may provide continuous geometric cues to guide cell distribution and organization, potentially influencing cell adhesion and spatial arrangement within the three-dimensional construct.

### 2.2. Rheological Properties of Bioinks

**Figure 3:**
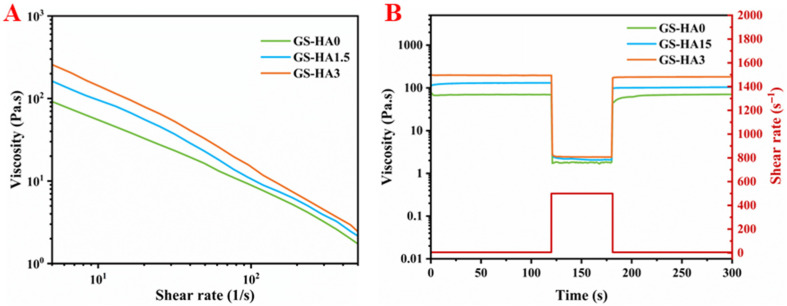
Rheological properties of GS-HA bioinks. (**A**) Viscosity as a function of shear rate. (**B**) Shear-recovery behavior under alternating shear rates.

As shown in Figure 3A, all prepared bioinks (GS-HA0, GS-HA1.5, and GS-HA3) exhibited typical shear-thinning behavior, in which viscosity decreased progressively with increasing shear rate. This property facilitates smooth extrusion during 3D printing and reduces the likelihood of nozzle clogging, which is a critical requirement for extrusion-based bioprinting to ensure stable material flow through the nozzle. In the shear-recovery tests (Figure 3B), all bioinks demonstrated a rapid viscosity recovery once the shear rate was reduced from high to low values, though the recovery did not reach the initial level completely. This partial recovery suggests good structural resilience and adequate stability for maintaining printed geometry after deposition, indicating reversible rearrangement of the polymer network and sufficient elasticity to preserve the printed shape. Such rheological characteristics are widely recognized as essential for achieving reliable printability and shape fidelity in hydrogel-based systems [30,31,32].

### 2.3. Swelling and Degradation Behavior

**Figure 4:**
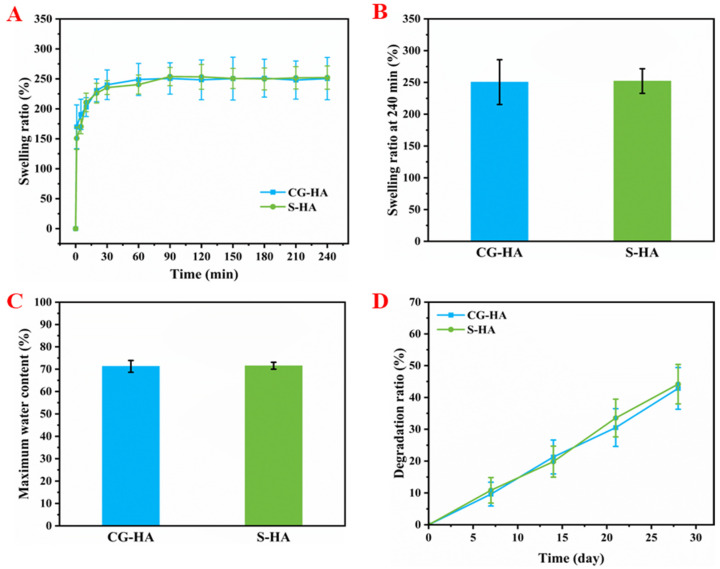
Swelling and degradation properties of CG-HA and S-HA scaffolds. (**A**) Swelling curves of the scaffolds. (**B**) Swelling ratios at 4 h. (**C**) Maximum water content. (**D**) Degradation curves over 28 days.

The swelling behavior of CG-HA and S-HA scaffolds displayed a pronounced early-stage water uptake, after which the swelling process progressively leveled off, reaching a steady state at around 90 min (Figure 4A). After 4 h of immersion, CG-HA and S-HA exhibited swelling ratios of 248.41 ± 18.89% and 255.02 ± 16.22%, respectively (Figure 4B). Correspondingly, their maximum water contents were 71.22 ± 1.63% and 71.78 ± 1.27%, respectively (Figure 4C), indicating similar hydrophilicity and water absorption capacities, suggesting a stable hydrogel network with controlled swelling behavior that is favorable for maintaining structural integrity while enabling efficient nutrient diffusion during cell culture.

The degradation behavior over 28 days is shown in Figure 4D. Both types of scaffolds exhibited a smooth and gradual mass loss profile without abrupt changes, suggesting stable degradation and good structural integrity throughout the incubation period, which is indicative of a well cross-linked network suitable for long-term in vitro culture. Moreover, such a moderate degradation profile may be advantageous for cartilage regeneration, as it can provide sustained mechanical support while gradually creating space for extracellular matrix (ECM) deposition.

### 2.4. Mechanical Properties

**Figure 5:**
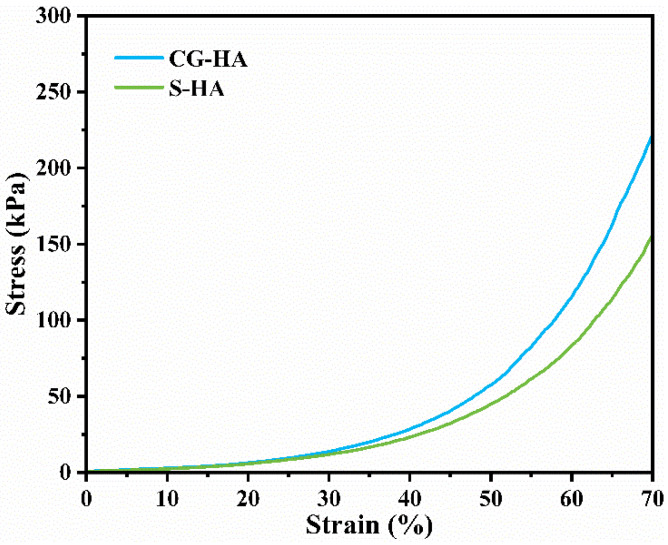
Stress–strain curves of CG-HA and S-H.

The mechanical behavior of CG-HA and S-HA was assessed under uniaxial compression. Figure 5 shows that both scaffolds displayed an initial elastic deformation region in their stress–strain profiles. The compressive strength and modulus of CG-HA (212.23 ± 36.18 kPa and 18.82 ± 5.33 kPa, respectively) were higher than those of the S-HA (151.26 ± 31.44 kPa and 18.72 ± 3.49 kPa, respectively). These results indicate that the continuous gradient design effectively improved the load-bearing capacity and overall mechanical stability of the scaffold, suggesting that gradual compositional transitions help reduce interfacial discontinuities and enhance the overall mechanical integrity of the construct. Notably, the compressive strength of CG-HA (212.23 ± 36.18 kPa) falls within the reported range for native articular cartilage (approximately 5–4000 kPa) [33], while its compressive modulus (18.82 ± 5.33 kPa) is slightly lower than the reported cartilage modulus range (approximately 20–7750 kPa) [34]. From a functional perspective, the present scaffold is designed to support early-stage cartilage regeneration and in vitro cartilage models related to articular cartilage defect repair, rather than to replicate the upper mechanical limits of native cartilage. In this context, CG-HA provides sufficient mechanical support to maintain structural integrity while offering a favorable microenvironment for bone marrow mesenchymal stem cell adhesion, proliferation, and differentiation. During cartilage regeneration, the scaffold acts as a temporary structural template facilitating extracellular matrix deposition and tissue formation. Therefore, although the mechanical properties of CG-HA are located at the lower end of the native cartilage spectrum, they are considered acceptable for its intended application.

### 2.5. Cell Viability and Proliferation

**Figure 6:**
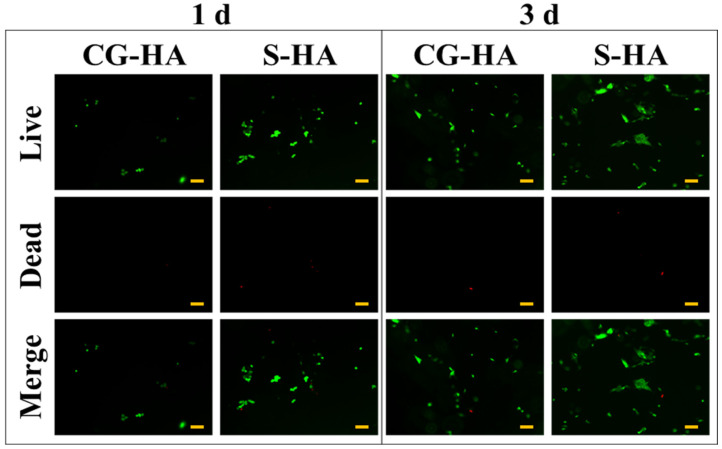
Representative fluorescence images showing BMSC viability on CG-HA and S-HA scaffolds after 1 and 3 days of culture. Live cells are shown in green and dead cells in red; scale bar = 100 μm.

Live/Dead staining revealed favorable cytocompatibility of BMSCs on both scaffold types (Figure 6). After 1 day of incubation, limited cell attachment was detected on the scaffold surfaces, with most cells maintaining a rounded morphology, indicating an early stage of adhesion. Following 3 days of culture, cell density and surface coverage increased noticeably, accompanied by the appearance of elongated and well-spread cell morphologies, reflecting progressive cell attachment and spreading. At both time points, only sporadic dead cells were observed.

**Figure 7:**
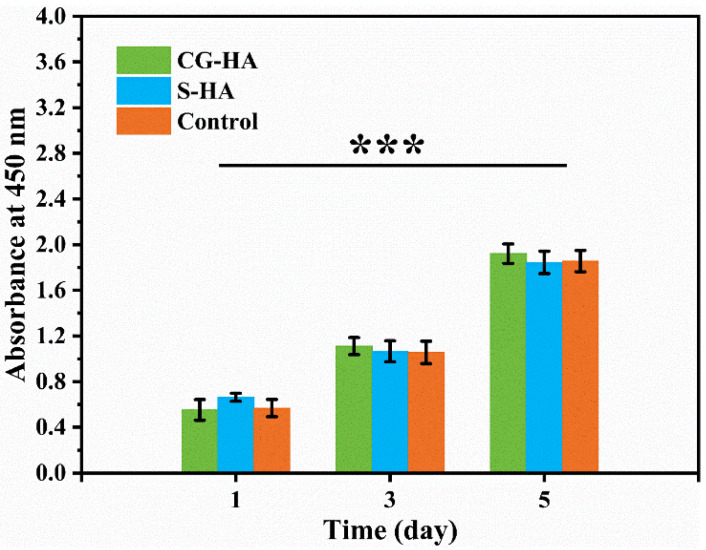
CCK-8 assay results at day 1, day 3, and day 5 (*** *p* < 0.001, *n* = 6).

The results of the Cell Counting Kit-8 (CCK-8) assay (Figure 7) further confirmed the cytocompatibility of the scaffolds. In this assay, the control group represented cells cultured in scaffold extract-free medium. The optical density (OD) values increased continuously from day 1 to day 5, indicating sustained cell proliferation over time.

Biological evaluation confirmed that both scaffold types exhibited good cytocompatibility. Live/Dead staining confirmed effective cell adhesion and high viability of BMSCs. In parallel, CCK-8 analysis demonstrated sustained proliferative activity of the cells over the culture period. Collectively, these results indicate that neither HA incorporation nor the employed preparation approach elicited detectable cytotoxicity. Moreover, the combination of a soft hydrogel matrix and a gradual HA gradient appears to provide a supportive environment for BMSC survival and growth.

### 2.6. Chondrogenic Differentiation in Scaffold Extracts

**Figure 8:**
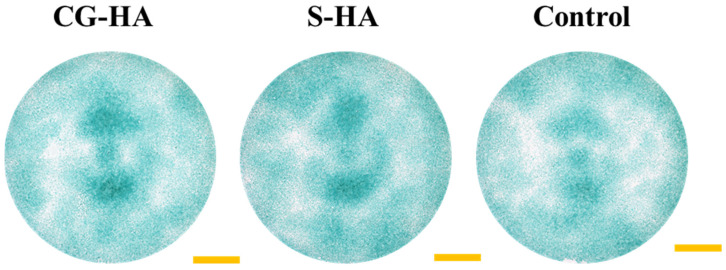
Alcian Blue staining of BMSCs cultured in extracts from CG-HA scaffolds, S-HA scaffolds, and blank control for 7 days (blue indicates glycosaminoglycan depositions; yellow scale bar: 3000 μm).

**Figure 9:**
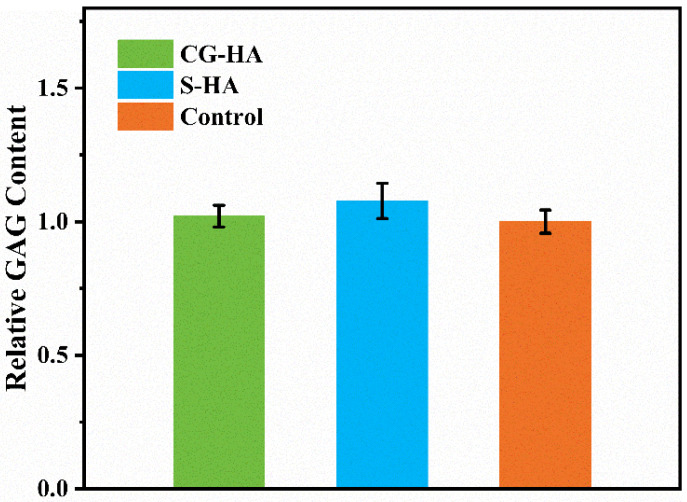
Semi-quantitative analysis of Alcian Blue staining intensity based on ImageJ 1.54p (values were normalized and presented as relative values).

To preliminarily assess the influence of scaffold components on BMSC chondrogenesis, cells were cultured for 7 days in extracts derived from the CG-HA scaffold, the S-HA scaffold, and a blank control, in which cells were maintained in chondrogenic induction medium without exposure to scaffold-derived extracts. Alcian Blue staining demonstrated evident blue staining across all three groups (Figure 8), reflecting effective chondrogenic induction accompanied by glycosaminoglycan (GAG) deposition. ImageJ 1.54p-assisted semi-quantitative evaluation of staining intensity (Figure 9) showed comparable levels among the groups, with no statistically significant differences detected (*p* > 0.05), indicating that the presence of HA in the composite formulation did not adversely affect the synthesis of cartilage-related extracellular matrix components and suggesting good biosafety with respect to BMSC chondrogenic differentiation under the tested conditions.

### 2.7. Limitations and Future Perspectives

Although the fabricated CG-HA scaffold successfully integrates a spatially curved architecture with a continuous HA concentration gradient and demonstrates acceptable mechanical and biological performance, several limitations remain. Native articular cartilage exhibits gradients not only in HA but also in other extracellular matrix components. Therefore, future studies should optimize the HA profile in combination with additional cartilage-associated components to construct a more biomimetic microenvironment. Moreover, the current biological evaluation is mainly based on extract-based assays, which do not capture spatial cell–matrix interactions. Future work will incorporate direct cell-laden scaffold cultures, together with zonal marker analysis of collagen type II (COL II), aggrecan (ACAN), and SRY-box transcription factor 9 (SOX9) under long-term or dynamic culture conditions. In addition, in vivo animal studies will be required to further validate the regenerative potential in clinically relevant cartilage defect models.

## 3. Conclusions

A spatially curved cartilage scaffold with a continuously decreasing HA concentration gradient from bottom to top was successfully fabricated using a dual-channel extrusion-based 3D printing strategy. The scaffold faithfully reproduced the designed curved architecture and showed stable swelling and degradation behavior. Compared with S-HA, CG-HA exhibited improved mechanical performance together with a more continuous compositional transition, suggesting that this design is better suited to mimic the mineral gradient distribution of native articular cartilage. Biological evaluation showed that CG-HA was cytocompatible and supported BMSCs’ adhesion and proliferation. Results from extract-based chondrogenic assays indicated that the incorporation of HA and its gradient distribution did not interfere with the synthesis of cartilage-related extracellular matrix components, confirming the biosafety of the composite system. Nevertheless, scaffold selection may still depend on specific application requirements, and S-HA can remain an appropriate option for simplified or layer-specific engineering designs. Further optimization of the HA gradient profile may allow more precise control over cellular spatial distribution and promote the development of zonal chondrocyte phenotypes along the vertical direction, thereby more closely recapitulating the layered organization of native articular cartilage.

## 4. Materials and Methods

### 4.1. Materials

The photoinitiator Irgacure 2959 was purchased from Shanghai Aladdin Biochemical Technology Co., Ltd. (Shanghai, China). SA and anhydrous calcium chloride (CaCl_2_) were sourced from Sinopharm Chemical Reagent Co., Ltd. (Shanghai, China). HA was provided by Shanghai Hualan Chemical Technology Co., Ltd. (Shanghai, China), while anhydrous ethanol was acquired from Shanghai Yien Chemical Technology Co., Ltd. (Shanghai, China). PBS was obtained from Wuhan Servicebio Technology Co., Ltd. (Wuhan, China). GelMA was synthesized according to our previously reported method [13].

### 4.2. Design of Scaffold

In articular cartilage, the transition from calcified cartilage tissue to the non-calcified superficial zone is characterized by a gradual decrease in mineral content rather than abrupt interfaces. To replicate this feature, the cartilage scaffold in this study was designed with a continuous compositional gradient of HA along the depth direction, integrated with a spatially curved morphology that conforms to the natural joint surface. This design aimed to enhance mechanical compatibility with subchondral bone while maintaining a biomimetic architecture suitable for cartilage regeneration.

For material selection, it was essential to ensure homogeneity across the gradient while allowing tunable compositional variations. GelMA was chosen as the primary matrix material due to its excellent biocompatibility and photocrosslinking capability, which facilitates 3D bioprinting with high precision [35]. SA was incorporated to improve the structural stability and printability of bioinks, while providing ionic crosslinking capability with CaCl_2_ [36,37]. HA nanoparticles were added to generate the mineral gradient, with the bottom layer containing the highest concentration and the top layer containing none, thus mimicking the natural calcified-to-hyaline cartilage transition.

With respect to the fabrication process, the gradient scaffold required precise spatial control over both geometry and material distribution. A dual-channel mixing extrusion strategy was employed to achieve continuous compositional variation. Specifically, two cartridges were used: one loaded with HA-containing bioink and the other with HA-free bioink. The two inks were simultaneously delivered into a mixing chamber, where they were homogenized before extrusion. By dynamically adjusting the feeding rates of the two cartridges, the HA concentration in the extruded filament could be finely controlled, thereby generating a continuous compositional gradient along the depth direction. The scaffold geometry was reconstructed using CAD modeling of the articular surface, and the curved structure was printed using coordinated motion control of the multi-axial system. Photocrosslinking was applied immediately after extrusion to stabilize GelMA, followed by ionic crosslinking in CaCl_2_ solution to further reinforce the SA network and enhance the overall mechanical strength of the construct.

To realize this fabrication strategy, a customized bioprinting platform integrating multi-axis motion and multi-material mixing. Material delivery was achieved using two independent cartridges coupled to a dual-channel mixing nozzle, with each cartridge driven by a stepper motor. The relative rotation speeds of the motors were dynamically regulated to precisely control the feeding rates of the two inks, thereby enabling adjustable HA content during printing. The motion module integrated X, Y, and Z linear stages together with A-axis tilting and C-axis rotation of the receiving platform, allowing deposition along spatially curved paths. Under this setup, planar positioning of the platform was controlled by the X–Y axes, vertical movement of the extrusion nozzle was governed by the Z axis, and coordinated rotations provided by the A and C axes ensured alignment with the curved deposition path. Given the temperature sensitivity of GelMA, a cooling module was integrated into the receiving platform to keep the ink temperature below 10 °C during deposition, thereby enhancing shape fidelity.

### 4.3. Fabrication of Scaffold

#### 4.3.1. Preparation of Bioinks

Three types of biomaterial inks were prepared for the fabrication of the gradient cartilage scaffold. Briefly, gelatin GelMA was dissolved in PBS under magnetic stirring until fully dissolved, followed by the addition of SA with thorough mixing. HA powder was subsequently incorporated and uniformly dispersed to obtain the bioinks. The detailed compositions and concentrations of all components are summarized in Table 1.

In addition, a 1% (*w*/*v*) photoinitiator solution was prepared by dissolving 1 g of photoinitiator in 100 mL of anhydrous ethanol, which was later used for the photocrosslinking process. A 5% (*w*/*v*) CaCl_2_ solution was prepared by dissolving 5 g of anhydrous CaCl_2_ in 100 mL of deionized water, which served as the ionic crosslinking agent for alginate after printing.

#### 4.3.2. 3D Printing of the Gradient Scaffold

Two types of HA gradient hydrogel scaffolds were fabricated using a dual-channel extrusion-based 3D bioprinting system, including a continuously graded HA hydrogel scaffold (CG-HA scaffold) and a stepwise-HA hydrogel scaffold (S-HA scaffold).

##### Fabrication of the CG-HA Scaffold

The CG-HA scaffold was fabricated using a dual-channel extrusion-based 3D bioprinting system. Two material reservoirs, referred to as cartridge A and cartridge B, were loaded with bioinks containing different HA concentrations. Specifically, cartridge A was filled with GS-HA3 bioink, while cartridge B contained GS-HA0 bioink.

During the printing process, cartridge A was initially activated to deposit the first layer. Subsequently, the feeding rate of cartridge A was gradually reduced, while cartridge B was simultaneously activated with an increasing feeding rate. The two bioinks were mixed in a sealed mixing chamber and extruded to fabricate the second to fourth layers, forming a transitional region with a progressively decreasing HA concentration. Thereafter, cartridge A was stopped, and cartridge B further increased its feeding rate to fabricate layers 5 through 14, corresponding to the HA-free region of the scaffold.

During printing, G-code–controlled multi-axis coordination (X, Y, A, and C) directed platform movement. Meanwhile, the dual-channel mixing extrusion nozzle was equipped with *Z*-axis motion control, enabling synchronized vertical adjustments during printing. The coordinated operation of the multi-axis platform and the Z-controllable dual-channel extrusion head enabled the fabrication of a cartilage scaffold with a spatially curved architecture and a continuous HA gradient distribution. After printing, the scaffold surface was uniformly sprayed with a photoinitiator solution prepared in anhydrous ethanol. The construct was then exposed to 365 nm ultraviolet light for 30 min to induce photocrosslinking of GelMA. Subsequently, the scaffold was immersed in a CaCl_2_ solution for 30 min to perform ionic crosslinking of alginate, further enhancing the mechanical stability and structural integrity of the scaffold.

##### Fabrication of the S-HA Scaffold

The S-HA scaffold was prepared as a stepwise-gradient control using discrete bioink compositions. GS-HA0, GS-HA1.5, and GS-HA3 bioinks were sequentially employed to fabricate different layers. Specifically, GS-HA0 was used to print the first layer, GS-HA1.5 was applied to fabricate layers 2–4, and GS-HA3 was used for layers 5–14. By discretely varying the bioink composition across layers, scaffolds with a stepwise HA distribution were obtained and served as the control group.

### 4.4. Rheological Characterization of Bioinks

The printability-related rheological properties of GS-HA0, GS-HA1.5, and GS-HA3 were characterized on a rotational rheometer (DHR-3, Waters, Milford, MA, USA). Viscosity responses as a function of shear rate were measured over a range of 1–500 s^−1^ to evaluate shear-thinning behavior. To mimic extrusion and post-deposition recovery during printing, a stepwise shear-recovery protocol was applied under alternating shear conditions. A stepwise shear protocol was applied, consisting of an initial low shear phase (5 s^−1^, 120 s), a subsequent high shear phase (500 s^−1^, 60 s), and a final recovery phase at 5 s^−1^ for an additional 120 s. All measurements were conducted under controlled conditions at a temperature of 25 °C, with three independent replicates performed for each bioink.

### 4.5. EDS Analysis

To semi-quantitatively characterize the spatial distribution of HA within CG-HA, EDS analysis was performed. The printed scaffolds were sectioned along the depth direction to expose different regions from the bottom to the top.

EDS elemental analysis was conducted at representative positions at different scaffold depths using a scanning electron microscope (SEM, SU-1510, Hitachi, Tokyo, Japan) equipped with an EDS detector. P/C ratios were calculated at each depth and used as a semi-quantitative indicator of the relative mineral content, with C originating mainly from the organic hydrogel matrix. The mean P/C value obtained from the bottom layer of the scaffold, corresponding to an HA concentration of 3% (*w*/*v*), was set as 1, and the P/C values at other depths were normalized accordingly. The depth-dependent variation in P/C values was analyzed to evaluate the gradient distribution of HA along the scaffold height.

### 4.6. Swelling Properties Analysis

The swelling behavior of CG-HA and S-HA was determined using a gravimetric method modified from our previous study [38]. Before measurements, CG-HA and S-HA scaffolds were placed in an oven and maintained at 27 °C until their mass reached a constant value, which was defined as the dry mass (*W_dry_*). The dehydrated scaffolds were then transferred into PBS and allowed to swell at ambient temperature. At predefined sampling intervals, samples were removed from the solution, and residual liquid on the surface was gently eliminated using filter paper. The mass of the swollen samples was immediately determined and denoted as the wet weight (*W_wet_*). Once the swelling equilibrium had been established, the equilibrium mass (*W_s_*) was obtained. Throughout the entire procedure, particular attention was paid to minimizing mechanical stress and avoiding any structural deformation of the scaffolds.

The swelling ratio (SR) was determined using the equation below:

(1) $$\mathrm{SR}\;(\%)=\frac{W_{\mathrm{wet}}-W_{\mathrm{dry}}}{W_{\mathrm{dry}}}\times 100\%$$

The maximum water content (MWC) was determined using the equation:

(2) $$\mathrm{MWC}\;(\%)=\frac{W_{s}-W_{\mathrm{dry}}}{W_{s}}\times 100\%$$

### 4.7. Degradation Properties

The in vitro degradation behavior of the scaffolds was examined by incubating the samples in PBS at 37 °C for 28 days, based on a previously reported method with slight adjustments [39]. The initial dry mass of each scaffold was recorded as *W*_1_. At designated time points, samples were removed from the solution, subjected to drying at 27 °C to a constant mass, followed by weighing to obtain *W*_2_. Throughout the degradation study, the PBS medium was refreshed weekly to maintain consistent conditions.

The degradation ratio (DR) was calculated according to the following equation:

(3) $$\mathrm{DR}\;(\%)=\frac{W_{1}-W_{2}}{W_{1}}\times 100\%$$

### 4.8. Mechanical Property Test

The printed scaffolds exhibited a bilaterally curved geometry, which made stable positioning during compression tests difficult. To achieve proper alignment and ensure uniform load transfer, custom polycaprolactone (PCL) fixtures were produced using fused deposition modeling to compensate for surface curvature and to create planar support interfaces, based on a previously reported design and testing approach [13]. The scaffold specimens were then subjected to uniaxial compression at a constant crosshead speed of 0.2 mm/min. Force–displacement data were continuously collected during testing and subsequently used to calculate the compressive modulus and assess mechanical performance.

### 4.9. In Vitro Cell Characterization

#### 4.9.1. Cell Culture

Biocompatibility of the CG-HA and S-HA was assessed through in vitro studies employing BMSCs derived from C57BL/6 mice (Cyagen Biosciences, Suzhou, China). The cells were maintained in α-MEM supplemented medium containing 10% fetal bovine serum and 1% penicillin–streptomycin. Cultivation was carried out in Petri dishes at 37 °C under a humidified atmosphere containing 5% CO_2_, with the culture medium replaced at two-day intervals. When cell confluence reached approximately 80%, subculture was performed using 0.25% trypsin–EDTA.

For subsequent biocompatibility assessment, the pre-cultured BMSCs were seeded directly onto the entire scaffold surface and incubated under the same culture conditions to evaluate cell adhesion, proliferation, and morphology on the gradient hydrogel structure.

#### 4.9.2. Cell Viability Assay

The CG-HA and S-HA underwent sterilization by immersion in 75% ethanol for 30 min, followed by ultraviolet (UV) exposure for 1 h. After sterilization, the scaffolds were washed three times with sterile PBS and incubated in freshly prepared culture medium for 2 h to facilitate ethanol removal. The treated scaffolds were then transferred into sterile culture plates prior to cell seeding.

BMSCs were applied to the scaffold surfaces at a density of 1 × 10^6^ cells/mL and allowed to attach under standard culture conditions (37 °C, 5% CO_2_) for 4 h. The culture medium was refreshed at two-day intervals throughout the incubation period.

Cell viability and distribution on the scaffold were evaluated using a Live/Dead cell staining kit (MX3012-500T, Maokangbio, Shanghai, China). According to the manufacturer’s instructions, 5 μL calcein-AM and 15 μL propidium iodide (PI) were simultaneously added to a total volume of 5 mL prepared from 10× dilution buffer to prepare the Live/Dead staining solution. After 15 min of incubation under dark conditions, fluorescence micrographs were obtained on days 1 and 3 using an inverted fluorescence microscope (LHM100CB-1, Nikon, Tokyo, Japan).

#### 4.9.3. Cell Proliferation Assay

To assess the influence of scaffold-derived components on BMSC proliferation, extract media were generated from the scaffolds prior to cell culture. Briefly, sterilized scaffolds were incubated in BMSC growth medium at 37 °C for 48 h to produce scaffold-conditioned media, while cells maintained in standard culture medium without scaffold exposure served as the blank control.

BMSCs were resuspended in the scaffold-conditioned medium at a concentration of 2 × 10^4^ cells/mL, and 100 μL of the cell suspension was dispensed into each well of a 96-well plate, followed by incubation at 37 °C under 5% CO_2_.

Cell proliferative activity was evaluated using a CCK-8 (Jiangsu KeyGen BioTech Co., Ltd., Nanjing, China) at 1, 3, and 5 days after seeding. At the designated time points, 10 μL of CCK-8 working solution was added to each well and allowed to react for 90 min. Absorbance was subsequently recorded at 450 nm using a microplate reader (Infinite 200Pro, Tecan Group Ltd., Männedorf, Switzerland), and the resulting optical density values were used to quantify cell proliferation.

#### 4.9.4. Chondrogenic Differentiation in Scaffold Extracts

To investigate the chondrogenic response induced by scaffold-derived components, a 6-well plate assay using scaffold extracts was performed. Sterilized scaffolds with HA and HA-free scaffolds were separately immersed in chondrogenic induction medium based on high-glucose DMEM, supplemented with fetal bovine serum (10%), penicillin–streptomycin (1%), L-proline (40 μg/mL), TGF-β3 (10 ng/mL), dexamethasone (0.1 μM), ITS premix (1%), and ascorbic acid-2-phosphate (1 μM) at 37 °C for 48 h to obtain the leachates.

BMSCs were plated in 24-well culture plates and allowed to adhere under standard incubation conditions (37 °C, 5% CO_2_). Once attachment was established, the culture medium was changed in accordance with the assigned experimental groups. Three groups were established: (i) a CG-HA extract group, in which cells were cultured with chondrogenic induction medium supplemented with extracts from CG-HA scaffolds; (ii) an S-HA extract group, cultured with chondrogenic induction medium containing extracts from S-HA scaffolds; and (iii) a blank control group, in which cells were maintained in chondrogenic induction medium without scaffold extracts. All groups were cultured for 7 days, and the corresponding media were refreshed three times per week.

On day 7, chondrogenic differentiation was assessed via Alcian Blue staining to detect glycosaminoglycan (GAG) deposition. Cells were gently washed twice with PBS, fixed in 4% paraformaldehyde at room temperature for 15 min, followed by two additional PBS rinses (3 min per wash), and subsequently incubated with Alcian Blue for a duration of 20 min. After thorough rinsing in tap water, images were collected under the same microscope settings.

For semi-quantitative analysis of GAG deposition, Alcian Blue-stained images were analyzed using ImageJ 1.54p software (NIH, Bethesda, MD, USA). All images were acquired under identical microscope settings to ensure comparability among different groups. During analysis, the images were first converted to 8-bit grayscale and processed under unified parameter conditions, with the same threshold settings applied to all samples. Regions of interest (ROIs) were then selected as circular areas of identical size centered at the center of each well in the culture plate, ensuring consistency of the analyzed regions among all samples. The integrated density of Alcian Blue staining within the selected ROIs was measured as a semi-quantitative indicator of staining intensity. The obtained values were then normalized to the mean value of the blank control group, which was set as 1, and expressed as relative staining intensity. Data for each group was obtained from at least three independent samples.

### 4.10. Statistical Analysis

All experimental results were processed using OriginPro 2022 software (OriginLab, Northampton, MA, USA). Results are expressed as mean ± standard deviation (SD) from no fewer than three independent experiments. Group comparisons were conducted using one-way analysis of variance (ANOVA), and a * *p* value below 0.05 was considered statistically significant.

## Figures and Tables

**Table 1:** The detailed compositions and concentrations of all components.

Biomaterial Inks	GelMA (*w*/*v*)	SA (*w*/*v*)	HA (*w*/*v*)	PBS (mL)
GS-HA0	10%	4%	0%	10
GS-HA1.5	10%	4%	1.5%	10
GS-HA3	10%	4%	3%	10

## Data Availability

The data presented in this study are openly available in article.
